# Potential Involvement of *Salmonella* Infection in Autoimmunity

**DOI:** 10.3390/pathogens8030096

**Published:** 2019-07-03

**Authors:** Zhanna Ktsoyan, Lyudmila Budaghyan, Marina Agababova, Armine Mnatsakanyan, Karine Arakelova, Zaruhi Gevorgyan, Anahit Sedrakyan, Alvard Hovhannisyan, Mkhitar Mkrtchyan, Magdalina Zakharyan, Arsen Arakelyan, Rustam Aminov

**Affiliations:** 1 Institute of Molecular Biology, National Academy of Sciences, Yerevan 0014, Armenia; 2 Institute of Cardiology after L.A. Hovhannisyan, Yerevan 0014, Armenia; 3 Nork Clinical Hospital of Infectious Diseases, Ministry of Health of the Republic of Armenia, Yerevan 0047, Armenia; 4 School of Medicine, Medical Sciences and Nutrition, University of Aberdeen, Aberdeen AB25 2ZD, UK

**Keywords:** *Salmonella*, salmonellosis, serotype, convalescent, autoantibody

## Abstract

In this work, we investigated the potential effects of nontyphoidal *Salmonella* infection on autoantibody (AA) formation. The titer and profiles of autoantibodies in the sera of patients with acute salmonellosis due to *Salmonella enterica* serovar Typhimurium (*S.* Typhimurium) or *Salmonella enterica* serovar Enteritidis (*S.* Enteritidis) infection, as well as in convalescent patients, were determined with indirect immunofluorescence. A significant increase of autoantibodies in acute diseases caused by both serotypes of *Salmonella* and during post infection by *S.* Enteritidis was detected. Antibody profile analysis by multivariate statistics revealed that this increase was non-specific and was not dependent on the infectious agent or disease stage. The results obtained suggest that nontyphoidal *Salmonella* infection contributes to the generation of autoantibodies and may play a role in autoimmune disease.

## 1. Introduction

In recent decades, the occurrences of autoimmune diseases have increased dramatically worldwide. These diseases are the result of the human body’s loss of self-tolerance and a mounting aggressive immune reaction towards itself [1]. Factors contributing to this phenomenon are not completely understood. The etiology of autoimmune diseases is multifactorial, and it is difficult to elucidate the exact role of genetic and environmental factors in the launch of an aggressive immune response against the body’s own cells [2]. While the genetic component of autoimmune disease undoubtedly plays a significant role in disease predisposition, the rapid increase in disease frequency over the last decades suggests the potential involvement of environmental factors. Infectious diseases caused by some bacteria, viruses, and protozoa are known to be inducers of elevated titers of antibodies towards annexin-V, prothrombin, laminin, anti-*Saccharomyces cerevisiae* antibodies (ASCA), antinuclear antibodies (ANA), and phospholipids [3]. These infections may constitute environmental factors that are involved in the initiation and promotion of autoimmune diseases in genetically predisposed individuals [1].

As mentioned above, epidemiological data suggest a steady increase in the instances of allergic and autoimmune diseases in the developed world [4]. At the same time, we are witnessing a substantial decrease in the frequency of infectious diseases, which, in light of the contradictory roles played by infections in autoimmunity [2], poses a question about the role of infections in autoimmune disease. Moreover, the apparent protective effects of some infections towards immune-mediated diseases add a new dimension to the problem [4]. Thus, the role of infectious agents relating to autoimmune or allergic disease is contradictory.

In animal models of autoimmune disease, environmental factors such as viral and bacterial infections could be considered to be the main contributing factors due to their ability to elicit strong immune and autoimmune reactions. In humans, the correlation between exposure to several pathogens and autoimmune disease is also well known. The most common infectious agents associated with reactive arthritis, for instance, are Gram-negative enteric pathogens such as the species of *Salmonella*, *Shigella*, *Yersinia*, and *Campylobacter*, and also *Chlamydia* [5]. Human leukocyte antigen (HLA)-B27, as the antigen presentation structure and the target for autoimmune effector cells, seems to play an important role in this process. A strong association between reactive arthritis and the aforementioned infections suggests the involvement of the autoimmune component in adaptive immunity towards infections [5]. In several animal experimental settings, infectious agents induced autoimmune diseases, some of which have clinical analogues [4]. In humans, it is difficult to prove a potential link between infectious disease and autoimmunity because of obvious ethical constraints as well as the fact that clinical manifestations of the latter may occur after a substantial period of subclinical autoimmune disease development [6]. A strong immune response to a pathogen is a must for the survival of the host, but after the pathogen(s) clearance it must be resolved to prevent chronic inflammation, which may lead to autoimmunity.

A variety of pathogens could be involved in the initiation of self-destructive immune responses. There is some supporting evidence for the association of certain infectious agents and infection-induced autoimmunity [1]. The autoimmunity could be induced by some facultative/obligate intracellular microbial agent(s) causing chronic infection, and some *Salmonella* spp. may be among them [7]. This autoimmune potential also depends on a particular host-microbe interaction as well. For instance, some *Salmonella* serovars such as *Salmonella enterica* serovar Typhimurium *(S.* Typhimurium) may have a commensal lifestyle in hosts other than humans. They can persist in the intestines of farm animals without causing disease [8,9].

Salmonellosis is one of the most frequently encountered gastrointestinal infections. It is mostly caused by nontyphoidal *Salmonella* such as *Salmonella enterica* serovars Typhimurium and Enteritidis, which are both significant foodborne pathogens worldwide [8]. Compared with other common foodborne pathogens, they have the most profound impact on human health [10,11,12]. The clinical manifestations include enterocolitis, which may be accompanied or followed by complications such as bacteremia and focal infections.

*Salmonella* is a unique pathogen in that it has evolved to avoid the immunological surveillance by, and to persist for long periods of time within, the host [13]. Salmonellosis has been mostly studied during the acute stage of infection, while chronic infections have been characterized to a lesser extent [14]. Despite that, the convalescent state is frequently encountered following acute *Salmonella* infection, but there is limited information available for this disease recovery stage. Multiple factors, including characteristic features of a pathogen and a host, as well as environmental factors, are involved in this process [15]. The convalescent state could be a risk factor in regards to the development of diseases with an inflammatory component, such as gastroenteritis or autoimmune and allergic diseases, because the prolonged chronic inflammation during this state may serve as a predisposing factor for such diseases. In exceptional cases, nontyphoidal *Salmonella* such as *S*. Typhimurium may cause systemic infection [16].

The gut environment has attracted considerable attention since it modulates the immune system and is therefore involved in immune-mediated disorders such as autoimmune diseases [17]. A growing body of evidence suggests that commensal microbiota and their metabolites are strong modulators of the immune system [18,19]. Alterations in the composition of gut microbial communities could be a risk factor in regards to the development of allergic and autoimmune diseases [20]. Gastrointestinal infections such as salmonellosis may substantially alter the gut microbiota composition, especially if the infection treatment includes antimicrobial therapy. 

Recently, we have investigated the impact of salmonellosis on the development of predisposition to allergic conditions [21]. In particular, we discovered a significantly elevated level of immunoglobulin E (IgE) in the sera of patients infected by *S.* Enteritidis while infections caused by *S*. Typhimurium had no such effect. In addition, we characterized the degree of dysbiosis in the salmonellosis patients to identify the potential effect of gut microbiota on increased IgE production. Interestingly, dysbiosis during salmonellosis can be ruled out as a potential factor contributing to the excessive production of systemic IgE during *S.* Enteritidis infection [21].

In this work, we explore the potential contribution of *Salmonella* infection on the initiation of autoimmune reactions. Investigation of the initial stages of this process is important because it may have a prognostic value for evaluating the risks of autoimmune disease development following *Salmonella* infection [3].

## 2. Materials and Methods

### 2.1. Sample Collection

The study groups included patients with salmonellosis admitted to the Nork Clinical Hospital of Infectious Diseases (Yerevan, Republic of Armenia) between 2015 and 2017. Patients with salmonellosis included in this study were from different regions of Armenia. A total of 76 patients with acute salmonellosis caused by *S.* Typhimurium (n = 14) and *S.* Enteritidis (n = 62) were enrolled in this study. The convalescent state (n = 7) was investigated in the subgroup of *S.* Enteritidis-infected patients, which were then followed up for eight months following the disease The control group consisted of 23 healthy medication-free volunteers. The gender and age distribution in the *S*. Typhimurium group was as follows: 10 males and 4 females; median age, 2.06 years old (interquartile range (IQR) 3.25–20.5, range 1.5–65.0). In the *S.* Enteritidis group, these values were as follows: 30 males and 32 females; median age, 5.42 years old (IQR 3.41–8.0, range 1.6–75.0). In the convalescent patients, the gender and age distribution were as follows: 4 males and 3 females; median age, 5.0 years old (IQR 4.42–6.42, range 4.0–8.0). The gender and age distribution in the control group was as follows: 10 males and 13 females; median age, 7.0 years old (IQR 4.5–23.0, range 3.0–39.0).

Patients selected for the study were not taking any type of medication, including antibiotics, before the hospital admission. The patients or guardians reported no previous allergies or autoimmune diseases. Fecal samples were taken on the first or second day of admission to the hospital. It should be emphasized that the blood samples for analysis for the presence of autoantibodies were taken on the following days: for patients infected with *S.* *Typhimurium* on day 11.23 ± 2.65 days and for S. Enteritidis-infected patients on day 8.69 ± 1.01. The samples of convalescent subjects were taken during a period of up to eight months (median = 5 ± 1.57 months) following hospital admission. For detoxification and rehydration in acute disease, all patients were receiving standard infusion therapy. At the time of discharge from the hospital, no presence of *Salmonella* had been detected in the feces or blood of any of the patients. 

### 2.2. Ethical Approval

All study subjects (or parents or guardians if a child) gave their written consent to give fecal and blood samples for the study. The study protocol was approved by the Ethics Committee of the Institute of Molecular Biology NAS RA (IORG number 0003427, assurance number FWA00015042, and IRB number 00004079).

### 2.3. Sample Processing and Bacterial Isolation

Diagnostics of salmonellosis was based on clinical presentations and laboratory analyses. Clinical presentations consistent with gastroenteritis were diarrhea, fever, nausea, vomiting, and abdominal cramps. Biochemical assays for *Salmonella* identification were: fermentation of glucose, negative urease reaction, lysine decarboxylase activity, negative indole test, H_2_S production, and fermentation of galactitol (dulcitol). Serotypes of *Salmonella* were determined using the standard White–Kauffmann–Le Minor scheme [22], with the use of commercially available polyvalent antisera for the flagellar (H) and lipopolysaccharide (O) antigens.

### 2.4. Detection of Autoantibodies

The profile of autoantibodies in the sera of patients with acute salmonellosis due to *S.* Typhimurium or *S.* Enteritidis infection was determined to estimate the potential autoimmune antibodies level during the disease using previously described methods [23]. Autoantibodies were detected by indirect immunofluorescence using cryosections of rat liver, kidneys, and stomach tissues according to the manufacturer’s protocols (BioSystems, Barcelona, Spain; https://www.biosystems.es). The sections were processed with non-fluorescent patient serum, separately using positive and negative controls, then sections were washed with phosphate-buffered saline (PBS) for 5 min. The sections were then incubated for 30 min with a fluorescein-isothiocianate (FITC) with Evan’s blue, followed by washing and viewing of the slides. The sections were examined using the fluorescence microscope Lyumam I-1. Positive and negative controls included in the kit (codes 44558 and 44648) were incorporated in the processing of sera samples to verify the assay performance. Samples were diluted in PBS before the assay (1:20, 1:40, and 1:80).

Detection of antibodies including ANA, anti-smooth muscle antibody (ASMA), anti-liver-kidney microsomal antibodies (LKM), antimitochondrial antibodies (AMA), and rheumatoid factor (RF) was performed as described previously [24,25]. The levels of ANA, ASMA, LKM, and AMA are reported in arbitrary units as either a ratio or units (U). The RF levels are expressed as mg/L. A negative titer, or normal range, was considered to be a background level of fluorescence with the sera diluted to 1:20 (or less than 20 U). Weakly positive results were from 1:20 to 1:30 dilution, and 20 to 30 positive results were greater than 1:30 dilution or greater than 30 U.

### 2.5. Detection of Concentration of Systemic Interleukins

The concentration of interleukin (IL)-10 and interferon gamma (IFN-γ) in the sera samples was determined with the commercially available enzyme-linked immunosorbent assay (ELISA) plates (Vector–Best, Russia). The plates were read on a Stat Fax 303 Reader (Awareness Technology Inc., USA). Calibration curves for determining the concentration of these cytokines in experimental samples were obtained using the standards included in the kits. Detection limits for these assays were: IL-10–1pg/mL and IFN-γ –2pg/mL. 

### 2.6. Statistical Analysis

A Statistical Analyses GraphPad Prism 5 (GraphPad Software Inc, San Diego, CA, USA) was used to perform the Mann–Whitney U-test to determine the statistical significance of differences among the groups studied. *P*-values <0.05 were considered to be statistically significant. Discriminant function analysis was carried out with IBM SPSS Statistics 19 (IBM, Armonk, NY, USA).

## 3. Results

### 3.1. Systemic Autoantibodies in *S.* *Typhimurium* and *S.* *Enteritidis* Infections

**Figure 1:**
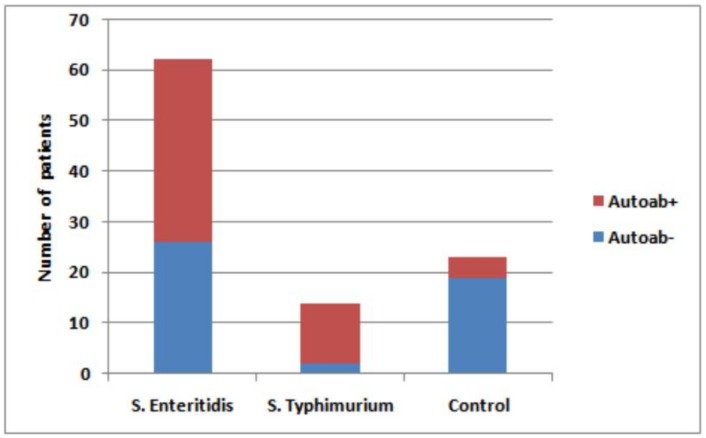
Presence (+) or absence (–) of tested autoantibodies in patients infected by *S.* Typhimurium (n = 14) or *S.* Enteritidis (n = 62) and in the control group (n = 23). The data represent patients that have one or more autoantibodies.

In this study, we measured the level of potential autoimmune antibodies in the blood of patients with acute salmonellosis caused by *S.* *Typhimurium* or *S.* Enteritidis, as well as in convalescent patients with the latter infection, in comparison with healthy control subjects. The presence of autoantibodies was revealed in 48 out of a total of 76 (63%) patients with acute salmonellosis (Figure 1). Specifically, autoantibodies were detected in 85.7% of patients with *S.* *Typhimurium* infection and in 58.1% with *S.* Enteritidis infection. 

**Figure 2:**
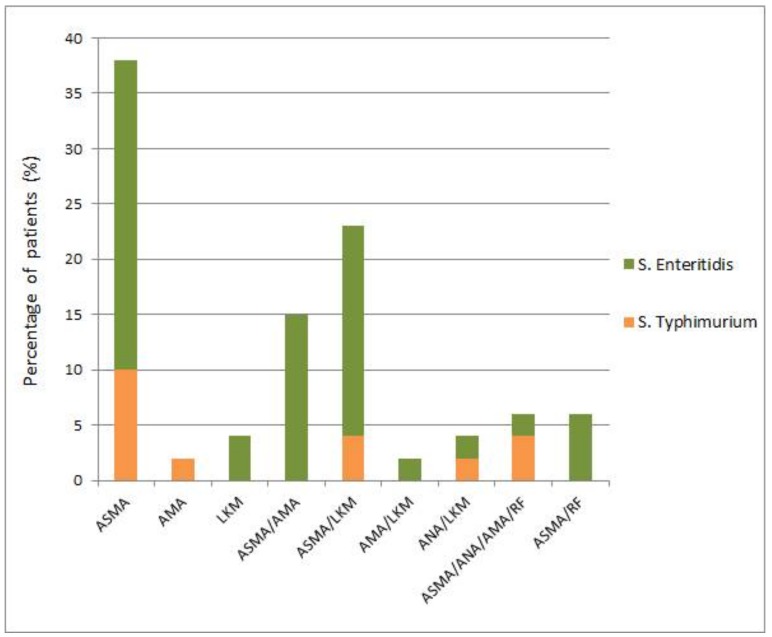
Prevalence of various autoantibodies in the subset of salmonellosis patients having autoantibodies. Percentage of patients with one autoantibody: anti-smooth muscle antibody (ASMA), 38% (column 1); antimitochondrial antibodies (AMA), 2% (column 2); and anti-liver-kidney microsomal antibodies (LKM), 4% (column 3). Patients with two autoantibodies: ASMA/AMA, 15% (column 4); ASMA/LKM, 23% (column 5); AMA/LKM, 2% (column 6); and antinuclear antibodies (ANA)/LKM, 4% (column 7). Patients with three antibodies and rheumatoid factor (RF) (ASMA/ANA/AMA/RF), 6% (column 8). Patients with ASMA and RF (ASMA/RF), 6% (column 9).

The prevalence of different autoantibodies was highly variable in the sera of patients. Among the mentioned total of 48 samples with autoantibodies, 42 (87.5%) displayed ASMA autoantibodies (Figure 2). In patients with ASMA autoantibodies, at least two autoantibodies were detected in 20 patients (41.7%). The high level of rheumatoid factor was found in six salmonellosis patients compared with the control group (88 mg/L vs. <20 mg/L, respectively).

Antibodies in the sera of salmonellosis patients were detected at dilutions 1:20, 1:40, and 1:80. Sera dilutions 1:20 and 1:40 produced the strongest signal for ASMA in a large number of samples (39 patients), while signals were very low for AMA (1 patient) and LKM (2 patients). Sera diluted to 1:80 was ASMA-reactive in only three patients. In the sera of the majority of patients, ANA (92%), AMA (77%), and LKM (67%) autoantibodies were not detected. At the same time, the ASMA autoantibodies were absent in only six patients (12.5%). In the healthy control group (n = 23), the 1:20 serum dilution was weakly positive in only four samples. However, it remained unclear whether the difference in the proportion of autoantibody-producing patients within each of four cohorts including healthy volunteers (23 total and four–positive for autoantibodies) and salmonellosis patients (76 total and 48–positive for autoantibodies) was infection-specific.

### 3.2. Multivariate Discriminant Function Analyses

To elucidate this, the discriminant analysis (DA) method of multidimensional statistics was applied, the dependent variables of which were three groups of patients that were positive for at least one antibody, and a control group. Predictors were the autoimmunity markers.

**Figure 3:**
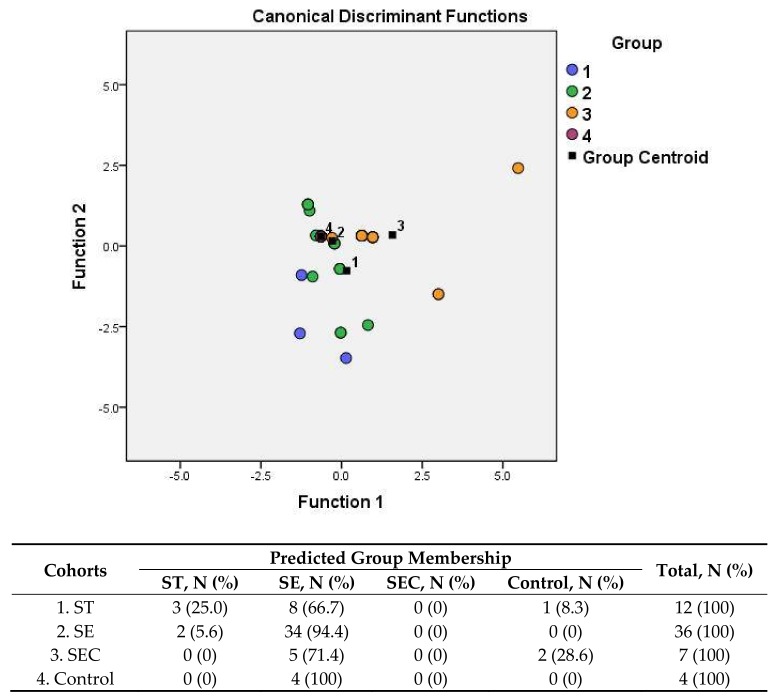
Scatterplot of the discriminant analysis (DA) model and the predicted group membership in the model based on the presence of various autoantibodies in the sera of salmonellosis patients and control subjects. The number of variables in the model is nine, and grouping consists of four groups. Root 1, two–discriminant functions one and two (1st and 2nd canonical roots). Cohorts: (1) *S*. Typhimurium-infected patients (ST), (2) *S.* Enteritidis-infected patients (SE), (3) SE convalescent patients (SEC), and (4) control subjects. Predictive accuracy of classification: 66.1% of the original group cases were correctly classified; Wilks’ λ = 0.550.

The results of DA analysis demonstrated that the groups cannot be reliably separated according to health status, disease-causing agents, or the disease stage (Figure 3). Thus, the conclusion is that the spectra of autoantibodies generated by these infections are non-specific and most likely are not driven by the sets of specific antigens present in infectious agents. 

### 3.3. Comparison of Autoantibody (AA) Profile of *S.* *Enteritidis* Infected Patients in Acute State and Convalescent

**Figure 4:**
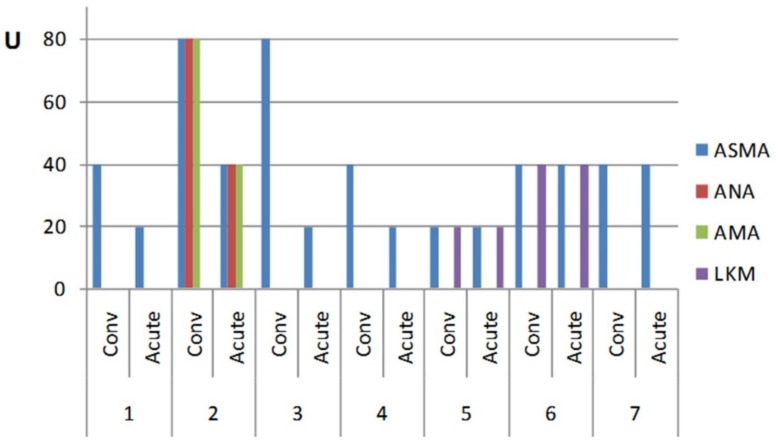
Autoantibody profile of *S.* Enteritidis-infected patients in acute disease and in convalescent state.

Interestingly, the convalescent stage following acute *S.* *Enteritidis* infection was also accompanied by its own specific autoantibody profile (Figure 4). In this group, in which some patients were followed up for a period of up to eight months (median = 5 ± 1.57 months), the levels of autoantibodies were two- to four-fold higher during the convalescent state compared with the acute disease in four salmonellosis patients, while in the other three patients the level was still persistently maintained above the basal level (Figure 4). Thus, recovery from the disease paradoxically resulted in an increased level of autoantibodies in some patients.

### 3.4. Levels of IL-10 and IFN-γ in All Cohorts

Next, with the use of ELISA, we determined the level of immunoregulatory cytokines, IL-10 and IFN-γ, in the systemic circulation of the subjects of all four cohorts. The cohorts tested for IL-10 included: patients with acute *S*. Typhimurium infection (n = 10), patients with acute *S.* Enteritidis infection (n = 51), post-*S.* Enteritidis infection patients in convalescent state (*n* = 7), and a healthy control group (n = 13).

**Figure 5:**
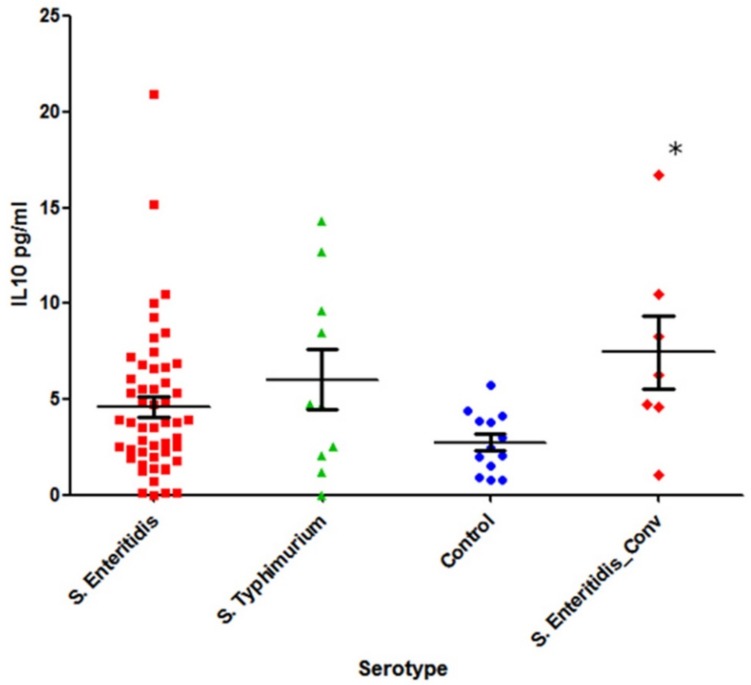
The level of IL-10 in the sera of patients infected with two serotypes of *Salmonella*, in acute disease and in convalescent state, and a control group (Mann–Whitney U-test): First column, *S.* Enteritidis-infected acute stage (n = 51); second column, *S.* Typhimurium-infected acute stage (n = 10); third column, healthy control group (n = 13); and fourth column, *S.* Enteritidis post infection stage (convalescent) (n = 7). * –*p* < 0.05.

Statistically significant differences between the concentration of IL-10 in *Salmonella*-infected patients and the healthy control group were seen only in the case of the *S.* Enteritidis convalescent group (*p* = 0.009). In the other two groups (acute disease due to *S.* Enteritidis or *S*. Typhimurium infection), the differences with the control group were not significant (*p* = 0.1 and *p* = 0.11, respectively, Figure 5).

**Figure 6:**
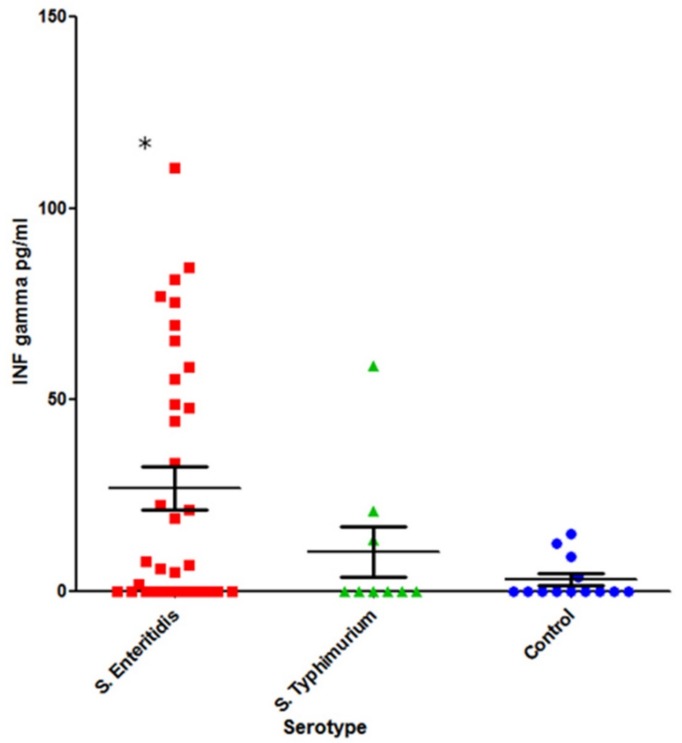
Concentration of IFN-γ in the sera of patients with acute salmonellosis and in a control group (Mann–Whitney U-test): First column, *S.* Enteritidis-infected (n = 35); second column, *S.* Typhimurium-infected (n = 9); and third column, healthy control group (n = 13). * –*p* < 0.05.

The cohorts tested for INF-γ included: *S*. Typhimurium-infected (n = 9) and *S.* Enteritidis-infected (n = 35) patients in acute disease stage and a healthy control group (n = 13) (Figure 6).

A statistically significant increase in systemic INF-γ was observed in *S.* Enteritidis-infected patients compared with the healthy control group (*p* = 0.02). In the case of *S*. Typhimurium infection, however, the difference was not statistically significant (*p* = 0.6).

We further investigated the levels of IL-10 and IFN-γ in the subgroups of patients who displayed elevated levels of autoantibodies. These subgroups were: (1) Patients with acute salmonellosis caused by *S*. Typhimurium with the presence of autoantibodies (n = 7); (2) patients with acute salmonellosis caused by *S.* Enteritidis with the presence of autoantibodies (n = 32 for IL-10 and n = 18 for IFN-γ); and (3) *S.* Enteritidis post-infection convalescent patients with the presence of autoantibodies (n = 7). The number of *S*. Typhimurium-infected patients in the acute disease stage without autoantibodies was too low (n = 2) to perform statistical analysis.

**Figure 7:**
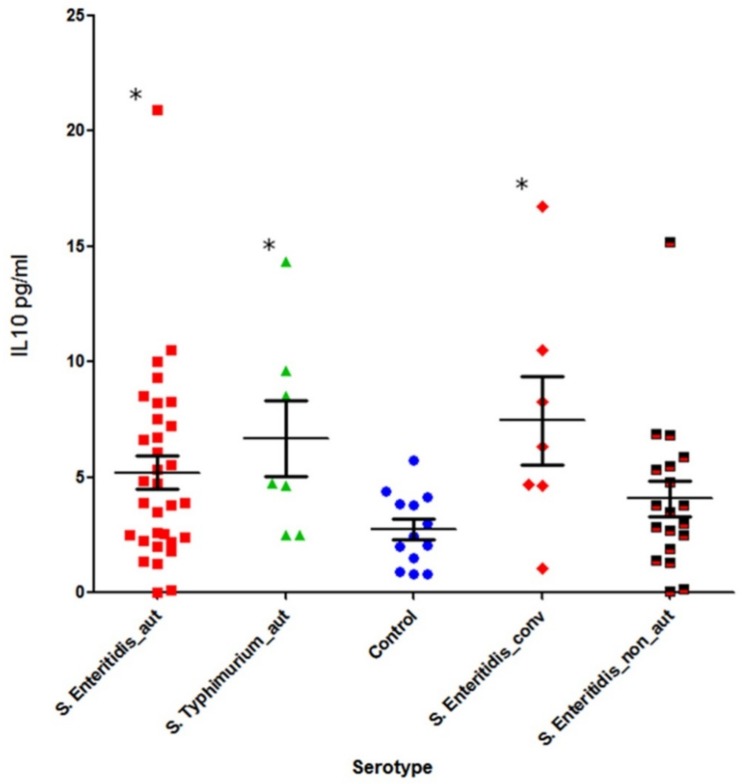
The level of systemic IL-10 in salmonellosis patients infected with two serotypes of *Salmonella* at different disease stages (Mann–Whitney U-test); First column, acute *S.* Enteritidis infection with autoantibodies (n = 32); second column, acute *S.* Typhimurium infection with autoantibodies (n = 7); third column, healthy control group (n = 13); fourth column, *S.* Enteritidis post-infection patients with autoantibodies (convalescent state, n = 7); and fifth column, *S.* Enteritidis acute stage without autoantibodies (n = 19). * –*p* < 0.05.

**Figure 8:**
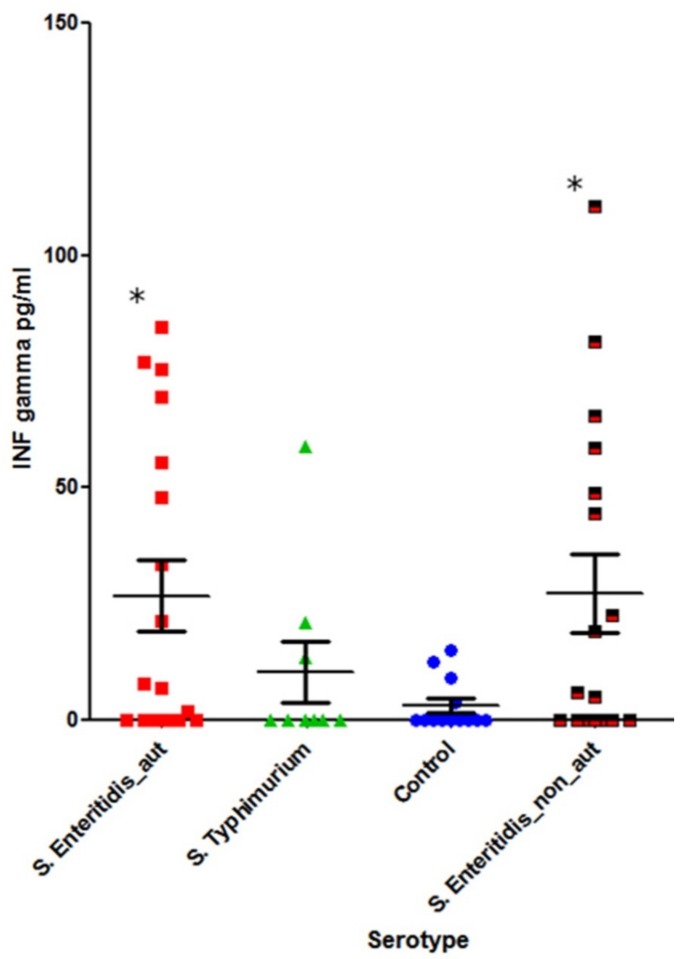
The level of systemic IFN-γ in salmonellosis patients infected with two *Salmonella* serotypes (Mann–Whitney U-test): First column, *S.* Enteritidis acute disease with autoantibodies (n = 18); second column, *S.* Typhimurium acute disease with autoantibodies (n = 7); third column, healthy control group (n = 13); and fourth column, *S.* Enteritidis acute stage without autoantibodies (n = 17) * –*p* < 0.05.

The levels of IL-10 and IFN-γ was also determined in salmonellosis patients with a normal level of autoantibodies (Figure 7 and Figure 8): *S.* Enteritidis-infected (IL-10 in 19 patients and IFN-γ in 17 patients), *S*. Typhimurium-infected (n = 2 for both IL10 and IFN-γ), and the control group (n = 13).

All groups showed a statistically significant increase of IL-10 compared with the control group, except for *S.* Enteritidis-caused acute disease without autoantibodies (fifth column) (*p* = 0.3) (Figure 7). The former groups included: acute *S.* Enteritidis infection with autoantibodies (*p* = 0.03), acute *S*. Typhimurium infection with autoantibodies (*p* = 0.01), and convalescent *S.* Enteritidis patients (*p* = 0.009).

A statistically significant increase of IFN-γ was noted for both *S.* Enteritidis acute disease groups (with and without autoantibodies) compared with the control group (*p* = 0.04), while *S*. Typhimurium infection (acute disease with autoantibodies) did not result in a statistically significant increase of IFN-γ compared with the control group (*p* = 0.6).

**Figure 9:**
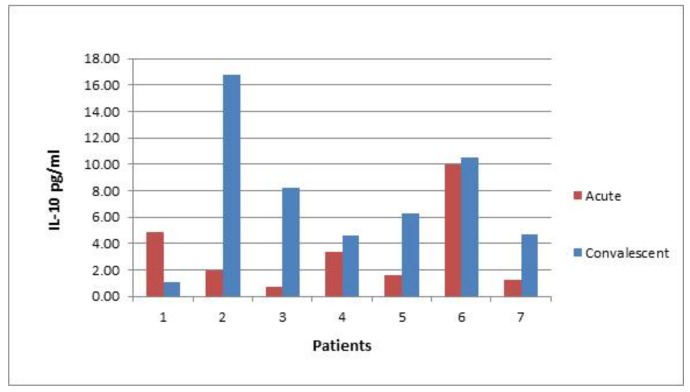
Individual dynamic of IL-10 levels in seven patients with acute infection by *S.* Enteritidis followed by convalescent stages.

In six out of seven convalescent patients post *S.* Enteritidis infection, the level of IL-10 was higher compared with the earlier acute stage of disease (Figure 9).

The levels of INF-γ in the post-infection group of *S.* Enteritidis infected patients (n = 7) were also tested. INF-γ was not detected in the serum of seven convalescent patients, and thus it is not shown. It should be noted that INF-γ was not detected in the serum of the same patients in the acute stage of disease.

## 4. Discussion

In this work, we investigated the effect of *Salmonella* infection on the possible induction of autoantibodies. For this, we determined the profiles and titer of autoantibodies during acute salmonellosis as well as in the post-salmonellosis convalescent period in comparison with control subjects. We found a significant increase of autoantibodies in acute disease caused by both serotypes of *Salmonella* and during post infection by *S.* Enteritidis. A comparative study of autoantibodies during the acute disease and convalescent stage revealed that the latter is characterized by significantly increased autoantibody titers. 

In general, the total percentage of salmonellosis and post-salmonellosis subjects displaying the presence of autoantibodies (63.15%) was substantially higher compared with the control cohort (17.39%). A certain low level of autoantibodies may be generated in healthy subjects due to a variety of reasons. In non-autoimmune individuals with various (bacterial, viral, parasitic, and rickettsial) infections, elevated titers of AAs to annexin-V, laminin, ASCA, ANA, and phospholipids were detected most often [3]. In all salmonellosis and post-salmonellosis groups, ASMA autoantibodies were the most prevalent. This autoantibody class is the serological hallmark of the majority of autoimmune diseases involving the liver [24,25]. Other autoantibodies may serve as predictors of disease outcome, but little is known about how specific they are as causes of autoimmunity induction. In general, a substantially increased titer of autoantibodies is a serological hallmark of most autoimmune diseases [26]. A commonly adopted view is that early detection of autoantibodies may help in predicting the development of autoimmune disease. In relation to this, we observed that autoantibody levels may persist and even increase in some patients long after the disease (Figure 4). The lack of normalization of the autoantibody level after a prolonged period of time could serve as another sign, which may call for further examination of these patients.

We also measured the systemic levels of two markers, IL-10 and IFN-γ, which may contribute to a better understanding of autoantibody induction during and after *Salmonella* infection. IL-10 plays a significant role in maintaining the gut homeostasis [27,28]. In infectious disease, it protects from excessive damage imposed by protective immune responses against it [29]. This may, however, lead to the persistence of infectious agents due to interference with innate and adaptive protective immunity. In the case of *S.* Typhimurium infection, for example, IL-10 may suppress the bactericidal response of macrophages against the infection [30]. Moreover, IL-10 may promote systemic dissemination of *S.* Typhimurium and impede pathogen clearance [31]. The potential pro-inflammatory properties of IL-10 should also be taken into consideration [32,33]. Our results demonstrated an interesting difference in IL-10 levels among the salmonellosis and post-salmonellosis cohorts that differed in autoantibody levels (Figure 5 and Figure 7). The subgroup of patients with elevated autoantibody levels tended to display elevated levels of IL-10 as well. This correlation is paradoxical and counterintuitive considering the tolerogenic effects of IL-10. However, more prolonged and broader organ exposure to the infectious agent mediated by IL-10 may have provoked less selective immune response targeting of the cell constituents, probably originating from damaged cells. The question, though, of why the level of IL-10 in these patients is higher remains open. These patients may have inherently possessed higher levels of IL-10, irrespective of the disease, which contributed to the sustained infectious agent exposure and subsequent autoantibody generation. The potential involvement of genetic components in the maintenance of higher IL-10 levels is indirectly supported by the measurement of IL-10 in convalescent patients, who still demonstrated elevated levels of IL-10 5 ± 1.57 months after hospital admission (Figure 9).

IFN-γ is vital for infection clearance, including *Salmonella* infections. IFN-γ response is primed or enhanced by certain commensal bacteria, and it provides both protection against, and clearance of, *Salmonella* infections [34,35]. Interestingly, we saw a significant induction of IFN-γ only in cases of acute infection caused by *S.* Enteritidis but not *S*. Typhimurium (Figure 6). At the same time, the level of IFN-γ was essentially independent from the autoantibody status (Figure 6 and Figure 8). 

In conclusion, our results suggest that infections caused by nontyphoidal *Salmonella* may induce a substantial level of autoantibodies. Moreover, these changes may persist long-term. It remains unknown, however, what the consequences of elevated levels of autoantibodies could be in terms of autoimmune disease development. Further studies are necessary to evaluate the risks associated with the induction of autoantibodies due to salmonellosis. 

## Abbreviations

AA, autoantibody; AMA, antimitochondrial antibodies; ANA, antinuclear antibodies; ASMA, anti-smooth muscle antibody; DA, discriminant analysis; ELISA, enzyme-linked immunosorbent assay; FITC, fluorescein-isothiocianate; HLA, human leukocyte antigen; IFN, γ-interferon gamma; IgE, immunoglobulin E; IL, interleukin; LKM, anti-liver-kidney microsomal antibodies; PBS, phosphate-buffered saline; RF, rheumatoid factor.

## References

[1] Ercolini A.M., Miller S.D. (2009). The role of infections in autoimmune disease. Clin. Exp. Immunol..

[2] Urs C., von Herrath M.G. (2005). Infections and Autoimmunity—Good or Bad?. J. Immunol..

[3] Berlin T., Zandman-Goddard G., Blank M., Matthias T., Pfeiffer S., Weis I., Toubi E., Singh S., Asherson R., Fraser A. (2007). Autoantibodies in nonautoimmune individuals during infections. Ann. N. Y. Acad. Sci..

[4] Bach J.-F. (2018). The hygiene hypothesis in autoimmunity: The role of pathogens and commensals. Nat. Rev. Immunol..

[5] Soloski M.J., Metcalf E.S. (2007). *Salmonella* as an Inducer of Autoimmunity. EcoSal Plus.

[6] Münz C., Lünemann J.D., Getts M.T., Miller S.D. (2009). Antiviral immune responses: Triggers of or triggered by autoimmunity?. Nat. Rev. Immunol..

[7] Tripathi M.K., Pratap C.B., Dixit V.K., Singh T.B., Shukla S.K., Jain A.K., Nath G. (2016). Ulcerative Colitis and Its Association with Salmonella Species. Interdiscip. Perspect. Infect. Dis..

[8] Boyen F., Haesebrouck F., Maes D., VanImmerseel F., Ducatelle R., Pasmans F. (2008). Non-typhoidal *Salmonella* infections in pigs: A closer look at epidemiology, pathogenesis and control. Vet. Microbiol..

[9] Kwag S.I., Bae D.H., Cho J.K., Lee H.S., Ku B.G., Kim B.H., Cho G.J., Lee Y.J. (2008). Characteristics of persistent *Salmonella* Enteritidis strains in two integrated broiler chicken operations of Korea. J. Vet. Med. Sci..

[10] Havelaar A.H., Kirk M.D., Torgerson P.R., Gibb H.J., Hald T., Lake R.J., Praet N., Bellinger D.C., de Silva N.R., Gargouri N. (2015). World Health Organization Global Estimates and Regional Comparisons of the Burden of Foodborne Disease in 2010. PLoS Med..

[11] Kirk M.D., Pires S.M., Black R.E., Caipo M., Crump J.A. (2015). Devleesschauwer, B.; Döpfer, D.; Fazil, A.; Fischer-Walker, C.L.; Hald, T.; et al. World Health Organization Estimates of the Global and Regional Disease Burden of 22 Foodborne Bacterial, Protozoal, and Viral Diseases, 2010: A Data Synthesis. PLoS Med..

[12] Scallan E., Hoekstra R.M., Mahon B.E., Jones T.F., Griffin P.M. (2015). An assessment of the human health impact of seven leading foodborne pathogens in the United States using disability adjusted life years. Epidemiol. Infect..

[13] Levine M.M., Robins-Browne R.M. (2012). Factors That Explain Excretion of Enteric Pathogens by Persons Without Diarrhea. Clin. Infect. Dis..

[14] Marzel A., Desai P.T., Goren A., Schorr Y.I., Nissan I., Porwollik S., Valinsky L., McClelland M., Rahav G., Gal-Mor O. (2016). Persistent Infections by Nontyphoidal *Salmonella* in Humans: Epidemiology and Genetics. Clin. Infect. Dis..

[15] Gunn J.S., Marshall J.M., Baker S., Dongol S., Charles R.C., Ryan E.T. (2014). *Salmonella* chronic carriage: Epidemiology, diagnosis, and gallbladder persistence. Trends Microbiol..

[16] Gilchrist J.J., MacLennan C.A., Hill A.V. (2015). Genetic susceptibility to invasive *Salmonella* disease. Nat. Rev. Immunol..

[17] Kelly D., Conway S., Aminov R.I. (2005). Commensal gut bacteria: Mechanisms of immune modulation. Trends Immunol..

[18] Kelly D., King T., Aminov R.I. (2007). Importance of microbial colonization of the gut in early life to the development of immunity. Mutat. Res..

[19] Mizuno M., Noto D., Kaga N., Chiba A., Miyake S. (2017). The dual role of short fatty acid chains in the pathogenesis of autoimmune disease models. PLoS ONE.

[20] Wu H.J., Wu E. (2012). The role of gut microbiota in immune homeostasis and autoimmunity. Gut Microbes.

[21] Ktsoyan Z.A., Mkrtchyan M.S., Zakharyan M.K., Mnatsakanyan A.A., Arakelova K.A., Gevorgyan Z.U., Ktsoyan L.A., Sedrakyan A.Ì., Hovhannisyan A.I., Ghazaryan K.A. (2015). Differential induction of total IgE by two *Salmonella* enterica serotypes. Front. Cell. Infect. Microbiol..

[22] Grimont P.A., Weill F.X. (2007). WHO Collaborating Centre for Reference and Research on *Salmonella*. Antigenic Formulae of the Salmonella Serovars.

[23] Romero-G’omez M., Wichmann I., Crespo J., Par´es A., Rodrigo L., Alvarez A., Diago M., Pons-Romero F., Sanchez-Munoz D., Aguilar-Reina J. (2004). Serum Immunological Profile in Patients with Chronic Autoimmune Cholestasis. Am. J. Gastroenterol..

[24] Blocka K. Anti-Smooth Muscle Antibody: Purpose, Risks, and Results—Healthline. https://www.healthline.com/health/anti-smooth-muscle-antibody.

[25] Zeman M.V., Hirschfield G.M. (2010). Autoantibodies and liver disease: Uses and abuses. Can. J. Gastroenterol..

[26] Fritzler M.J. (2008). Challenges to the use of autoantibodies as predictors of disease onset, diagnosis and outcomes. Autoimmun. Rev..

[27] Kuhn R., Lohler J., Rennick D., Rajewsky K., Muller W. (1993). Interleukin-10-deficient mice develop chronic enterocolitis. Cell.

[28] Rennick D.M., Fort M.M., Davidson N.J. (1997). Studies with IL-10−/−mice: An overview. J. Leukoc. Biol..

[29] Mege J.L., Meghari S., Honstettre A., Capo C., Raoult D. (2006). The two faces of interleukin 10 in human infectious diseases. Lancet Infect. Dis..

[30] Lee K.-S., Jeong E.-S., Heo S.-H., Seo J.-H., Jeong D.-G., Choi Y.-K. (2011). IL-10 suppresses bactericidal response of macrophages against *Salmonella* Typhimurium. J. Microbiol..

[31] Salazar G.A., Peñaloza H.F., Pardo-Roa C. (2017). Interleukin-10 Production by T and B Cells Is a Key Factor to Promote Systemic *Salmonella* enterica Serovar Typhimurium Infection in Mice. Front. Immunol..

[32] Lauw F.N., Pajkrt D., Hack C.E., Kurimoto M., van Deventer S.J., van der Poll T. (2000). Proinflammatory effects of IL-10 during human endotoxemia. J. Immunol..

[33] Tilg H., van Montfrans C., van den Ende A., Kaser A., van Deventer S.J., Schreiber S., Gregor M., Ludwiczek O., Rutgeerts P., Gasche C. (2002). Treatment of Crohn’s disease with recombinant human interleukin 10 induces the proinflammatory cytokine interferon gamma. Gut.

[34] Ost K.S., Round J.L. (2017). A Few Good Commensals: Gut Microbes Use IFN-g to Fight *Salmonella*. Immunity.

[35] Thiemann S., Smit N., Roy U., Lesker T.R., Gálvez E.J.C., Helmecke J., Basic M., Bleich A., Goodman A.L., Kalinke U. (2017). Enhancement of IFN-γ Production by Distinct Commensals Ameliorates *Salmonella*-Induced Disease. Cell Host Microbe.

